# Cohesion of Sister Chromosome Termini during the Early Stages of Sporulation in Bacillus subtilis

**DOI:** 10.1128/JB.00296-20

**Published:** 2020-09-23

**Authors:** Clare Willis, Jeff Errington, Ling Juan Wu

**Affiliations:** a Centre for Bacterial Cell Biology, Biosciences Institute, Newcastle University, Newcastle-upon-Tyne, United Kingdom; Ohio State University

**Keywords:** axial filament, chromosome cohesion, chromosome segregation, endospore formation, *Bacillus subtilis*

## Abstract

During sporulation of Bacillus subtilis, the cell cycle is reorganized to generate separated prespore and mother cell compartments, each containing a single fully replicated chromosome. The process begins with reorganization of the nucleoid to form an elongated structure, the axial filament, in which the two chromosome origins are attached to opposite cell poles, with the remainder of the DNA stretched between these sites. When the cell then divides asymmetrically, the division septum closes around the chromosome destined for the smaller prespore, trapping the origin-proximal third of the chromosome in the prespore. A translocation pore is assembled through which a DNA transporter, SpoIIIE/FtsK, transfers the bulk of the chromosome to complete the segregation process. Although the mechanisms involved in attaching origin regions to the cell poles are quite well understood, little is known about other aspects of axial filament morphology. We have studied the behavior of the terminus region of the chromosome during sporulation using time-lapse imaging of wild-type and mutant cells. The results suggest that the elongated structure involves cohesion of the terminus regions of the sister chromosomes and that this cohesion is resolved when the termini reach the asymmetric septum or translocation pore. Possible mechanisms and roles of cohesion and resolution are discussed.

**IMPORTANCE** Endospore formation in Firmicutes bacteria provides one of the most highly resistant life forms on earth. During the early stages of endospore formation, the cell cycle is reorganized so that exactly two fully replicated chromosomes are generated, before the cell divides asymmetrically to generate the prespore and mother cell compartments that are critical for the developmental process. Decades ago, it was discovered that just prior to asymmetrical division the two chromosomes enter an unusual elongated configuration called the axial filament. This paper provides new insights into the nature of the axial filament structure and suggests that cohesion of the normally separated sister chromosome termini plays an important role in axial filament formation.

## Abstract (precis)

Endospore formation in Firmicutes bacteria provides one of the most highly resistant life forms on earth. During the early stages of endospore formation, the cell cycle is reorganized so that exactly two fully replicated chromosomes are generated, before the cell divides asymmetrically to generate the prespore and mother cell compartments that are critical for the developmental process. Decades ago, it was discovered that just prior to asymmetrical division the two chromosomes enter an unusual elongated configuration called the axial filament. This paper provides new insights into the nature of the axial filament structure and suggests that cohesion of the normally separated sister chromosome termini plays an important role in axial filament formation.

## INTRODUCTION

Spore formation in Bacillus subtilis serves as an interesting model for the study of chromosome segregation. During vegetative growth, a copy of the chromosome must be segregated to each of the daughter cells (1). This is also true for sporulation; however, during sporulation, the two daughter cells comprise a large mother cell and a smaller, asymmetrically localized prespore cell. Prior to asymmetric division, the sister chromosomes change their confirmation to form a structure called the axial filament, which stretches across the cell from pole to pole (2–7). The origin regions of each chromosome are anchored at each cell pole through protein complexes involving RacA, Soj, Spo0J, and MinD (8–10). This ensures that about 30% of one chromosome is trapped in the prespore when the cell forms a division septum, asymmetrically, near one pole of the cell (11). To segregate the remaining 70% of the chromosome destined for the prespore, a translocase protein called SpoIIIE assembles in the division septum and actively pumps the DNA into the prespore (12–14).

This work is concerned with characterizing the behavior of the terminus region during chromosome translocation into the prespore in early stages of sporulation. The terminus region of the chromosome in B. subtilis comprises, approximately, the region between 152° and 187° of the chromosome, in which the nine replication termination Ter sites are located (15–19). This region is presumably the last region to be translocated into the prespore during sporulation. The hexameric SpoIIIE translocase assembles around double-stranded DNA, and it is thought that separate complexes translocate each chromosomal arm into the prespore (20–23). It has been proposed that the SpoIIIE complexes on each arm disassemble and make a pore for transferring the terminus region, which arrives last at the septum (24, 25).

The terminus regions of both chromosomes are located at midcell in the axial filament and, once the asymmetric septum has formed, are both located in the mother cell (3, 26). To study how the terminus is segregated and when it is translocated into the prespore, we used time-lapse microscopy combined with a microfluidic system. Using a fluorescent *tetOR* system, we found that there are frequently two distinct terminus foci prior to and during chromosome translocation, contrary to previous results (26). These terminus foci are closely apposed and move together toward the asymmetric septum during chromosome translocation, as if a cohesive force connects the sister termini. This cohesion appears to be broken soon after the termini reach the polar septum, presumably coinciding with translocation of the terminus belonging to the prespore chromosome across the septum. Possible mechanisms and roles of terminus cohesion are discussed.

## RESULTS

### Apparent cohesive behavior of sister chromosome termini during prespore chromosome translocation.

A *tetOR* FROS system was used to visualize the chromosome termini during prespore translocation (3, 26–30). A plasmid containing an array of approximately 240 *tetO* sequences was constructed for single crossover chromosome integration downstream from the *dacC* gene at 171°. To visualize the array, a TetR-mCherry fusion protein was expressed from the *ycgO* locus (297°). Previous research showed that operator arrays based on *tetO* or *lacO* systems can cause “roadblocks” for the DNA replication machinery (31, 32). However, in more recent work, *tetO120* arrays were shown not to cause large deviations in nucleoid length or in the number of origins present in a cell when a weak constitutive promoter (P*_ftsW_*) was used for expression of TetR-mCherry (29). We chose to use the same P*_ftsW_-tetR-mCherry* construct in our work, combined with a *tetO240* array. Neither growth rates nor terminus frequencies were detectably affected by the *tetOR-mCh* compared with control strains (see Fig. S1 in the supplemental material).

To visualize the membrane in time-lapse experiments, the *tetOR-mCh* fusion was combined with a construct constitutively expressing fluorescently labeled WALP23 (30). WALP23 is an artificial helix of 23 residues, consisting of mostly alanine and leucine amino acids (33). It inserts into the cell membrane as a transmembrane helix (34) and, when fused to a fluorescent protein, labels the cell membrane (35), allowing the division septum as well as the cell outline to be visualized. Cells entering sporulation were imaged using the CellASIC Onix microfluidic system in which live cells were immobilized under an elastic ceiling and supplied with constant medium flow, maintaining the cells at 32°C. Under these conditions, cells grew and sporulated more slowly than in batch culture, possibly because of reduced aeration.

**FIG 1:**
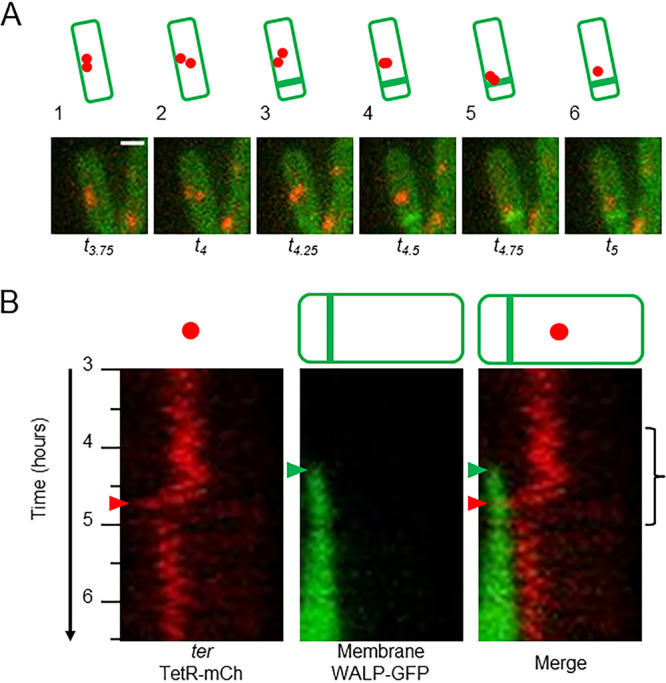
Time-lapse imaging of *ter* localization in a wild-type strain during sporulation. (A) Static images at 15-min intervals taken from a representative cell from time-lapse imaging every 3 min using the microfluidic system of strain CRW447 (*tetOR-mCh WALP23-gfp*). Images show an overlay of the red (terminus focus) and green (membrane) channels. Schematic representations of the cells are shown above the images. Bar, 1 μm. (B) Kymograms for the red (terminus) and green (membrane) channels and for the merged signals are shown, created from the same cell as shown in panel A and depicting intensities through the length of the cell from *t*_3_ to *t*_6.5_. The time of asymmetric septation (green arrowheads) and terminus translocation (red arrowheads) are shown. The bracket indicates the time period from which the static images in panel A were taken. Corresponding Movie S1 is shown in the supplemental material.

A derivative of the wild-type strain 168CA harboring a terminal *tetOR-mCh* and a *WALP23-gfp* construct, which we called the *ter* localization strain, was analyzed in time-lapse experiments with imaging every 3 min. Figure 1 and the corresponding time-lapse movie (see Movie S1 in the supplemental material) show a typical cell from 10 events captured. The imaging (stills in panel A and associated kymogram in panel B) revealed two closely localized foci (red) in the middle of the cell between *t*_3.75_ and *t*_4.25_ (Fig. 1A, snapshots 1 to 3). Asymmetric septation occurred at around *t*_4.5_ (visible by the strong green signal and indicated by the green arrowheads in the kymogram), and shortly afterwards the terminus signal, as either one focus (e.g., frame 6) or as two closely localized foci (e.g., frame 5), moved toward the septum (note that the asymmetric septum appeared much brighter than the rest of the cell, presumably because of the double membrane and perpendicular arrangement relative to the focal plane). The sharp diagonal in the red channel of the kymogram between *t*_4.5_ and *t*_5_ signals a relatively rapid processive movement of the labeled termini toward the asymmetric septum. The red arrowheads highlight the time point at which the terminus foci arrived at the septum. Subsequently, only one terminus focus was visible in the time-lapse analysis, localized on the mother cell side of the septum; the other terminus had presumably been translocated into the prespore. Previous work showed that proteins such as green fluorescent protein (GFP) are stripped from the chromosome as the locus passes through the SpoIIIE translocase (36). These results support the idea that both termini of the sister chromosomes move together toward the asymmetric septum, shortly after its formation. Nine other cells were analyzed and showed a similar pattern of terminus movement to that described above (see Fig. S2 and S3 in the supplemental material). In each cell, occasional brief separation of the two terminus foci was observed, but both termini remained overlapping or close together as they moved progressively toward the asymmetric septum. The main differences between the cells were in the positioning of the mother cell terminus after its arrival at the asymmetric septum (or after the prespore terminus entered the prespore); in some cases, the mother cell terminus remained close to the asymmetric septum (cells 1, 5, 7, 8, and 9), while in the other 4 cells the terminus moved away from the septum.

A fluorescently tagged version of the replication termination protein (RTP) (RTP-GFP), expressed from the native *rtp* locus, was analyzed by snapshot microscopy during bulk culture sporulation (see Fig. S4 in the supplemental material). RTP binds DNA at the termination site and has been shown to be a good marker for the position of the chromosome terminus (37). The analysis showed that some cells had two terminus foci in the axial filament at stage I of sporulation and following asymmetric septation at stages IIi and IIii, analogously to the *ter* localization strain described above. The strain containing the RTP-GFP protein was unfortunately too faint for time-lapse imaging.

### Measurement of the rate of chromosome translocation.

**FIG 2:**
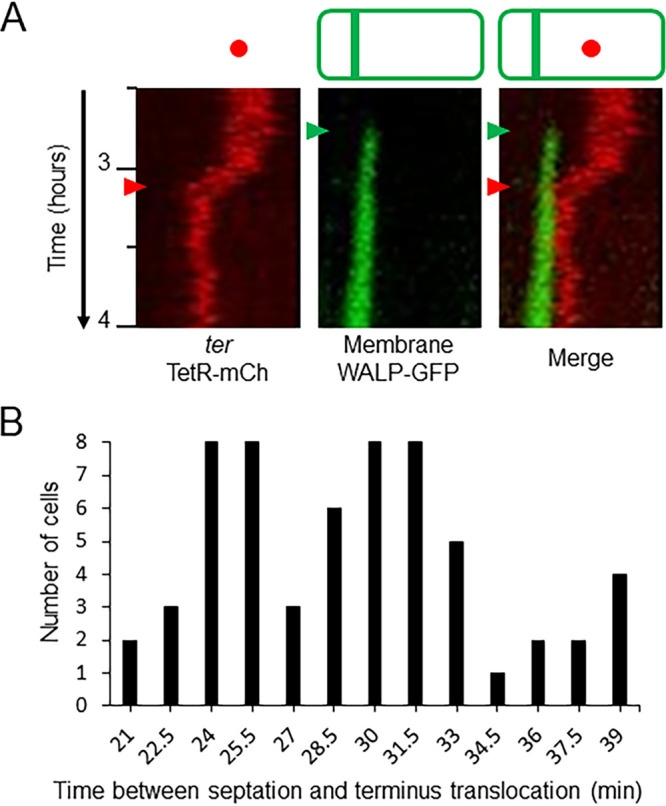
Time-lapse imaging with 90-s intervals to follow terminus movement. Three independent time-lapse experiments using the microfluidic system were carried out for strain CRW447 (*tetOR-mCh WALP23-gfp*) in sporulation, imaging every 90 s. (A) Kymograms from a time-lapse experiment between *t*_2.5_ and *t*_4_ for one representative cell, showing red (terminus) and green (membrane) channels alongside a merge of both kymograms (corresponding Movie S2 is shown in the supplemental material). The times of asymmetric septation (green arrowheads) and terminus translocation (red arrowheads) are highlighted. (B) Bar chart showing the distribution of times measured between septation and the terminus translocation in 60 cells.

To measure the rate of chromosome translocation, the imaging of the *ter* localization strain was repeated at 90-s intervals. The more frequent imaging resulted in a reduction in sporulation frequency, presumably due to increased photodamage (38). In one representative field (*n* > 250 cells) for time-lapse imaging at 90-s intervals, the sporulation efficiency (judged by asymmetric septum formation) was 10%, compared with 33% in the experiments with 3-min intervals. Nonetheless, kymograms for the cells imaged at 90-s intervals showed the same pattern of movement of the terminus as that described above (Fig. 2A; see Movie S2 in the supplemental material). The sister chromosomal termini moved together toward the asymmetric septum shortly after it was formed, appearing as one focus in some frames and as two separated foci in other frames. Following movement to the asymmetric septum, one terminus was assumed to be translocated into the prespore (see Fig. S5 in the supplemental material). In general, the movement of the termini appeared smoother in the 90-s time-lapse experiments than in the 3-min time-lapse experiments.

The rate of translocation was measured in 60 cells in total, 20 each from three independent time-lapse experiments. The frames between asymmetric septation and terminus translocation were counted to determine the time taken to translocate the remaining two-thirds of the prespore chromosome. These 60 data points are displayed graphically in Fig. 2B. The moment of septation was defined as the frame in which the septum stretched the full width of the cell, while the point of terminus translocation was defined as the first frame in which a terminus focus was adjacent to or overlapping with the asymmetric septum. Calculation of the rate assumed that translocation began immediately after the septum was formed and that translocation of each arm of DNA was carried out in parallel by separate SpoIIIE complexes. The amount of DNA trapped in the prespore is approximately 1.2 Mbp (11, 39, 40) of the 4.2-Mbp chromosome (41); therefore, the amount of DNA to be translocated is ∼3 Mbp, roughly 1.5 Mbp per chromosome arm. The time taken between septation and terminus translocation had a mean average of 29.2 ± 4.8 min. Assuming symmetrical translocation and one SpoIIIE complex per arm, this corresponded to a rate of SpoIIIE-mediated translocation of ∼860 bp/s (at 32°C). This is similar to values derived from previous *in vivo* studies. Burton et al. (22) measured a transfer time of 20 min at 37°C (equivalent to 1,250 bp/s), based on differential timing of expression of σ^F^-dependent reporter genes at origin and terminus regions. Ptacin et al. (42) reported 500 ± 80 bp/s at 30°C through the use of a fluorescent DNA dye to measure the rate of increase of fluorescent intensity in the prespore and, accordingly, the rate of translocation of the chromosome into the prespore.

### Terminus movement is dependent on SpoIIIE.

Terminus movement was assumed to be driven by the translocation of DNA by SpoIIIE. To check this, a *spoIIIE36* mutation that abolishes DNA translocation (39, 40, 43), was introduced into the *ter* localization strain. In contrast to the results described above, no terminus movement was detected upon imaging of this strain, as shown in the kymograms of a representative cell in Fig. S6 and corresponding Movie S3 in the supplemental material; the two terminal foci, again staying very close to each other, remained at an approximately midcell position throughout the time-lapse experiment. This pattern was observed in 10 of 10 cells analyzed, confirming that movement of the chromosome termini toward the prespore is dependent on functional SpoIIIE, as suggested previously (26). Moreover, separation of the apparent terminus cohesion may also be dependent on SpoIIIE.

### Terminus translocation into the prespore.

In the *ter* localization experiments described above, TetR-mCh foci did not appear within the prespore, presumably because the TetR-mCh proteins are stripped off the *tetO* binding sites as the DNA travels through the hexameric SpoIIIE ring (36). To confirm that the terminus was indeed translocated into the prespore, strains were designed to allow the terminus to be visualized also after it entered the prespore. To do this, an additional copy of *tetR* was positioned at −7°, near the replication origin, under the control of a prespore-specific promoter, P*_spoIIQ_*. This second prespore-specific pool of fluorescent TetR protein could bind to the 171° *tetO* array only after the terminus entered into the prespore. Two versions of *tetR* constructs at −7° were made, *ori-tetR-mCh* and *ori-tetR-gfp*; they were separately introduced into the *ter* localization strain.

**FIG 3:**
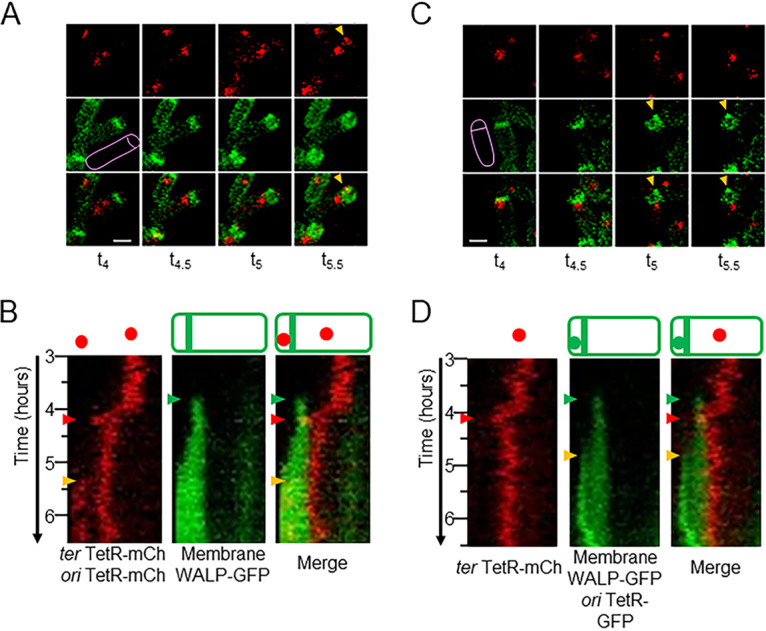
Visualization of terminus translocation into the prespore. Time-lapse imaging with 3-min intervals using the microfluidic system of strains CRW540 (*tetOR-mCh WALP23-gfp ori-tetR-mCh*) (A and B) and CRW509 (*tetOR-mCh WALP23-gfp ori-tetR-gfp*) (C and D) during sporulation. CRW540 has red signals from two fusions and one green signal, while CRW509 has green signals from two fusions and one red signal. (A) Static images of a representative cell from a time-lapse analysis of CRW540, each snapshot 30 min apart. Snapshots show the red channel (terminus in mother cell and prespore), the green channel (membrane), and an overlay of the two channels. (B) Kymograms for the cell shown in panel A between *t*_3_ and *t*_6.5_ (corresponding Movie S4 is in the supplemental material), showing red (terminus in mother cell and prespore) and green (membrane) channels, alongside a merge of both kymograms. (C) Static images of a representative cell from a time-lapse analysis of CRW509, each snapshot 30 min apart. Snapshots show red channel (terminus in mother cell), green channel (membrane and terminus in prespore), and an overlay of the two channels. (D) Kymograms for the cell shown in panel C between *t*_3_ and *t*_6.5_ (corresponding Movie S5 is in the supplemental material), showing red (terminus in mother cell) and green (membrane and terminus in prespore) channels, alongside a merge of both kymograms. Bar (A and C), 1 μm. (B and D) Times of asymmetric septation (green arrowheads) and terminus translocation (red arrowheads) are shown. (A to D) The appearance of prespore-specific TetR signal is indicated (orange arrowheads).

Strains carrying either the additional *ori-tetR-mCh* or *ori-tetR-gfp* constructs were imaged in time-lapse experiments, with representative snapshots and kymograms shown in Fig. 3 (see also Movies S4 and S5 in the supplemental material). For both strains, terminus foci were detected in the prespore (yellow arrowheads in Fig. 3), which implied that the 171° *tetO* array was indeed bound by prespore-specific fluorescent TetR proteins, confirming that terminus translocation did occur. In both cases, a delay was evident between the termini in the mother cell reaching the septum (Fig. 3, red arrowheads) and the appearance of a terminus focus in the prespore (Fig. 3, orange arrowheads). In the *ori-tetR-mCh* strain, a delay of 53 ± 7 min was measured from 10 cells. With the *ori-tetR-gfp* strain, the green signals of the septum and the terminus foci were distinguishable only based on shape and position, but the timings appeared qualitatively similar.

### Clear terminus association in disporic mutant cells.

Results thus far established that the termini of the sister chromosomes remain in close proximity while moving in parallel from midcell to the asymmetric septum, after which the prespore chromosome terminus is transferred through the septum. The delay observed between the termini arriving at the asymmetric septum and a focus appearing in the prespore could be due to the slow folding or long maturation time of the fluorescent TetR proteins in the prespore or to the extra time needed for SpoIIIE to remove the TetR proteins from the DNA to allow translocation. Alternatively, it could be indicative of a delay in translocation of the terminus into the prespore. To test this further, localization of the *ter* region was analyzed in a *spoIIGA* “disporic” sporulation mutant (44, 45). This mutant lacks σ^E^ activity, the sigma factor required for mother cell-specific gene expression (46, 47). Normally, σ^E^ activation blocks the formation of a second polar septum (45, 48). In its absence, a second asymmetric septum forms at the “mother cell” pole, generating a “disporic” phenotype in which prespores form sequentially at the two cell poles, with a large compartment in between, and the prespore and mother cell chromosomes are translocated sequentially into the opposing polar compartments (49).

**FIG 4:**
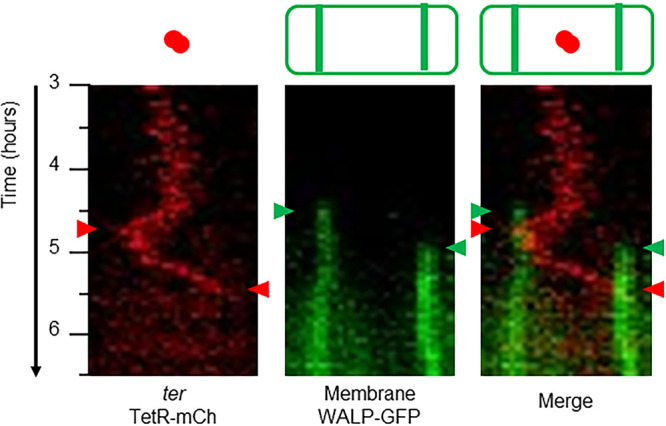
Kymograms from a time-lapse analysis of a *spoIIGA* mutant. Time-lapse imaging at 30-min intervals using the microfluidic system was carried out for strain CRW595 (*tetOR-mCh WALP23-gfp spoIIGA*) during sporulation. Kymograms are shown for a representative cell from the time-lapse analysis between *t*_3_ and *t*_6.5_, showing red (terminus) and green (membrane) channels, alongside a merge of both kymograms. Formation of each asymmetric septum (green arrowheads) and translocation of each terminus (red arrowheads) are shown. Corresponding Movie S6 is shown in the supplemental material.

Time-lapse imaging of the *spoIIGA* mutant confirmed that the asymmetric septa form sequentially, with a gap of 40.5 ± 10.3 min. As expected, both termini moved together toward the first septum (Fig. 4; see also Movie S6 in the supplemental material), as in wild-type cells (Fig. 1). However, after the second septum formed, the remaining terminus focus moved rapidly back across the cell, disappearing when it reached the second septum, presumably due to translocation into the second prespore. No mCherry foci were visible in the central compartment thereafter. This pattern was seen for all of the 10 cells analyzed. The time between the assumed translocation of the terminus at the first septum and formation of the second septum was 19 ± 10 min (*n* = 10 cells) and, following the formation of the second septum, the second terminus was translocated in 27 ± 10 min (*n* = 10 cells). In comparison, the first terminus was translocated in 22 ± 10 min (*n* = 10 cells). Thus, the rates of translocation of the two termini were roughly similar, even though the second terminus had twice the distance to travel. This was perhaps unsurprising, given that the same amount of DNA was translocated by each SpoIIIE translocase complex. These observations provided strong evidence that the two chromosome termini are closely connected during translocation from the midcell to the prespore septum during sporulation and that this connection is broken sometime after arrival at the septum.

### Relative timing of replication termination and terminus translocation.

It seemed possible that the apparent terminus cohesion could be due to a very late block in chromosome replication at a region beyond 171° where the *tetO* array was located, resulting in the replicated sister chromosomes being connected by the unreplicated region at the terminus, even though the fluorescently labeled region had been duplicated (see Discussion). To examine this possibility, DNA samples from sporulating cultures of wild-type 168CA growing at 37°C were probed by quantitative PCR (qPCR) to track the progression of replication through three sites in the terminus region, 166°, 172°, and 179°. The 166° site is located close to the *dif* site; the 172° site is close to the *rtp* gene and the TerI and TerII sites, within the region identified as having a stalled replication fork (50); the 179° site is within the *terC* region previously described (51).

Each locus was compared to that of a marker in the origin (*ori*) region, and the *ori*/*ter* ratios were normalized to spore DNA, which is known to contain one intact chromosome and so is assumed to have an *ori*/*ter* ratio of 1 (52, 53). An *ori*/*ter* ratio of 2 would imply twice as many origins as termini and thus that replication was ongoing. The *ori*/*ter* ratio should decrease as sporulation proceeds, as ongoing rounds of replication terminate and no new rounds are initiated.

**FIG 5:**
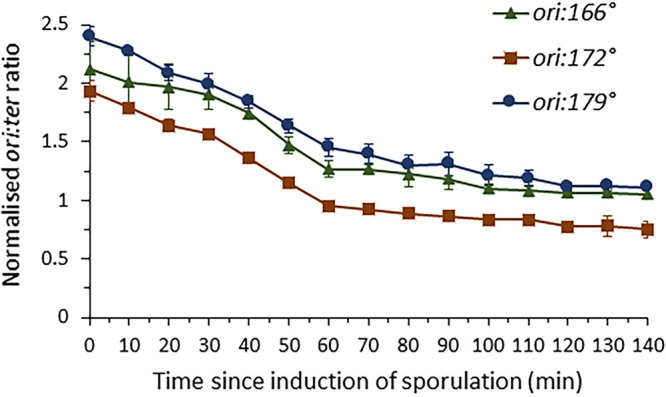
Quantitative PCR to assess progression of replication through sporulation. Strain CRW1 (168CA) was induced to sporulate at *T*0 minutes. Samples were taken throughout sporulation at 10-min intervals, and chromosomal DNAs of these were used in qPCR. Primers at *ori*, *ter-166°*, *ter-172°*, and *ter-179°* were used, and the *ori/ter* ratios for each of these sites (*ori*:*166°*, *ori*:*172°*, and *ori*:*179°*) were measured and plotted in the graph.

The *ori*/*ter* ratios indeed decreased in the first 60 min for each of the three terminus sites, suggesting that rounds of replication completed gradually between time zero minutes (*T*_0_) and *T*_60_ (Fig. 5). The differences in the absolute *ori*/*ter* values for each of the three sites are not likely to be meaningful and are most likely due to variation in primer binding efficiencies. Notably, however, all three plots showed similar rates of decrease during the first 60 min. After 60 min, the gradients became shallower, indicating that most cells had completed replication by this point. These results showed that replication at all three sites in the terminus region was completed by approximately 60 min. Microscopic examination of cells from the same cultures was used to examine the timing of asymmetric septation. Consistent with previous results (54), asymmetric septa only began to be detected from about *T*_75_ onward. These results suggested that replication was completed in the vast majority of cells before asymmetric septation began.

As a more direct way to examine the timing of completion of replication, we employed a *dnaN-gfp* fusion. DnaN is the sliding clamp of the replication machinery in B. subtilis, which accumulates behind the replication forks and which mostly dissociates from replication sites in the absence of elongation (55), providing us with a marker for the completion of chromosome replication. Snapshots and kymograms from time-lapse imaging of the *dnaN-gfp ter* localization strain are shown in Fig. S7 in the supplemental material. The DnaN-GFP and WALP23-GFP (membrane) signals (both green) were distinguishable by their characteristic localizations. A single DnaN-GFP focus was observed at midcell in the early stages of the time-lapse experiment (Fig. S7A, frames 1 and 2). For the representative cell shown, the DnaN-GFP focus dispersed about 2 h before asymmetric septation (Fig. S7B, yellow arrows). The relative timing between the dissociation of the DnaN-GFP focus and asymmetric septation was measured in 20 cells, giving a mean average of 109 ± 39 min (see Fig. S8 in the supplemental material). It seemed that the timing between these events varied greatly from cell to cell but was in general long, compared with the time from asymmetric septation to terminus translocation, which was measured at 31 ± 8 min in 20 cells (Fig. S8; comparable to “29.2 ± 4.8 min” obtained). Based on both these and the qPCR results, it appears that chromosome replication is usually completed well before the onset of prespore chromosome translocation.

## DISCUSSION

### SpoIIIE-dependent comigration of replicated sister chromosome termini during sporulation.

We set out to characterize the movement and dynamics of the chromosomal termini during chromosome segregation in sporulation in B. subtilis. To summarize a series of experiments, the duplicated terminus foci appeared to remain close to each other and to move in parallel from the midcell position toward the asymmetric septum during chromosome translocation, despite only one chromosome being destined for the prespore. After arriving at the polar septum, the apparent association between the chromosomal termini was resolved, and one terminus was translocated into the prespore. The terminus belonging to the mother cell remained in the mother cell after reaching the asymmetric septum.

The localization of both chromosomal termini close to the asymmetric septum at about the time of terminus translocation is consistent with the published results of Bogush et al. (26). Our results also support their proposal that segregation of the terminus is dependent on SpoIIIE, as movement of the termini from midcell was abolished in a *spoIIIE36* translocation-defective mutant. However, in contrast to the results presented by Bogush et al. (26), in which a single terminus focus was reported, in our experiments (using either RTP or a *tetO* array at 171°) we readily resolved two distinct, though very close, terminus foci prior to and throughout chromosome translocation. The difference may reflect different chromosomal sites (*pelB* locus at 174° in Bogush et al. [26], and *tetO* at 171° in this work) for the fluorescent markers or improved spatial resolution with our more recent imaging systems. The fact that the two termini remained in close proximity for an extended period in the nontransferring *spoIIIE* mutant mother cells, as well as during chromosome translocation in *spoIIE^+^* cells, is consistent with the idea that the sister termini cohere in the early stages of sporulation.

### Terminus decohesion occurs sometime after arrival at the polar septum.

By expressing fluorescent TetR protein in the prespore, we confirmed that the extreme terminus was translocated into the prespore. The absence of a prespore terminus focus in the experiments in which TetR was expressed only in the mother cell supported previous reports that proteins are stripped off the DNA as SpoIIIE-mediated chromosome translocation occurs (36). The surprisingly long delay observed between the terminus arriving at the asymmetric septum and a focus appearing within the prespore most likely reflects a delay in expression and subsequent production of fluorescent TetR proteins in the prespore. Formation of DNA-bound fluorescent TetR in the prespore would require activation of the prespore-specific P*_spoIIQ_* promoter, which occurs only after completion of the asymmetric septum, transcription, translation, and then maturation of the fluorescent protein. The mCherry protein is known to mature relatively slowly (40 min at 30°C). It is also possible that the last chromosomal segment requires a longer time to be translocated and released into the prespore or that the TetR array affects this process.

Observations of a *spoIIG* disporic mutant provided important insights into features of terminus behavior. Initially, the paired foci moved rapidly toward the first polar septum, as for wild-type cells. Then, after a short delay, roughly equivalent to the delay between disappearance of the first terminus into the prespore and formation of the second asymmetric septum, the terminus label, now presumably corresponding to a single terminus, moved processively toward the second septum, where it soon disappeared, presumably being translocated to the second polar cell. This behavior is most consistent with a model in which the two terminus regions are initially connected, but the connection is resolved when the termini reach the asymmetric septum. The second terminus can remain close to or move away from the septum in wild-type cells or translocate across the cell in disporic mutants, presumably driven by SpoIIIE activity at the distal septum. The rapid disappearance of the terminus after reaching the second polar septum is consistent with it being translocated and the TetR protein stripped from the DNA. These findings are contrary to the previous suggestion that the terminus is only translocated toward the end of engulfment, as disporic mutants do not engulf (26, 56). It is interesting that after translocation of the prespore terminus in wild-type cells, the behavior of the mother cell terminus was variable, suggesting that constraints on its location become relaxed after the sister chromosomes are completely resolved.

### Possible roles and mechanisms of terminus cohesion.

**FIG 6:**
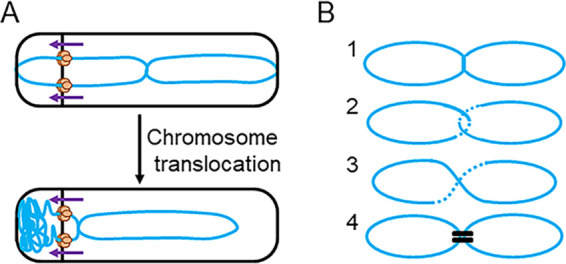
Diagrams of terminus cohesion. (A) Diagram depicting the cohesion at the terminus regions of the sister chromosomes (blue) during chromosome translocation (purple arrows). SpoIIIE translocase complexes are shown in orange, assembled in hexamers around each arm of the double-stranded DNA. (B) Diagram showing the 4 models of terminus cohesion: 1, late block in DNA replication; 2, chromosome catenation; 3, chromosome dimerization; and 4, protein bridging. Sister chromosomes represented in blue.

Comigration of the two termini during chromosome translocation suggests they are cohered in some way (Fig. 6A). The molecular basis for this apparent “terminus cohesion” will require further investigation but at this stage we can imagine at least four models to explain the effect (Fig. 6B). In model 1, “late block in replication,” a small region of DNA in the terminus region could remain unreplicated, and this would link the chromosomes together until replication was completed. The idea of delayed replication of the terminus region was interesting because it was potentially consistent with old experiments in which the terminus region was identified by incorporation of radiolabeled nucleotides (50). Our imaging showed two terminus foci during chromosome translocation, which indicated that the 171° locus had been replicated, but this did not rule out a late replication block elsewhere in the terminus region. However, two lines of evidence appear to support rejecting this model: first, qPCR analyses at three terminus-proximal sites all showed uniform completion of replication by 60 min of sporulation, before asymmetric septation begins; and second, a labeled DnaN-GFP protein consistently showed complete dissociation from the terminus region, indicating that replication was no longer ongoing, well before asymmetric septation.

Model 2, “catenation” (Fig. 6B2), proposes that catenanes in the terminus region could be responsible for terminus cohesion. Precatenanes form behind replication forks during the normal process of replication and, once replication is complete, precatenanes are converted to catenanes, physical links between daughter and parent DNA strands (57, 58). Type II topoisomerase enzymes (DNA gyrase or topoisomerase IV [Topo IV]) resolve catenanes (59, 60). In Escherichia coli, the DNA translocase protein FtsK activates Topo IV at division sites, so that catenation is resolved before the cell divides (61–63). Our imaging showed that terminus cohesion is resolved close to the asymmetric septum where the SpoIIIE DNA translocase, a homologue of FtsK (64–66), localizes. SpoIIIE could plausibly activate topoisomerase enzymes in the vicinity of the asymmetric septum, in an analogous way to FtsK and Topo IV in E. coli.

According to the third model, “chromosome dimerization” (Fig. 6B3), an odd number of recombination events between chromosomes leads to dimerization. These covalent linkages between sister chromosomes are thought normally to be resolved by recombinases, namely, by XerC and XerD in E. coli, which have homologues, RipX and CodV, in Bacillus subtilis (67, 68). The recombinases mediate strand exchange at the *dif* site (166°, in the terminus region) to resolve sister chromosomes before segregation (69, 70). SpoIIIE and another FtsK homologue, SftA, have been implicated in chromosome dimer resolution during vegetative growth in B. subtilis through positioning of the *dif* sites of sister chromosomes (69, 71). In sporulation, chromosome dimerization could hold the sister chromosomes together, followed by resolution at the asymmetric septum, potentially assisted by SpoIIIE. A limitation of this model would be that in exponentially growing cells, only 15% of cells form chromosome dimers (69), whereas all cells analyzed here (>60 cells) showed the phenotype consistent with cohesion at the terminus. Therefore, if chromosome dimerization was responsible for cohesion, a mechanism would be needed to ensure that an uneven number of recombination events always occurred in all cells during the final round of replication preceding sporulation. Also, it does not appear that *xerC* (*ripX*) and or *xerD* (*codV*) mutants have the impact on sporulation predicted by this model (72).

Finally, the “protein bridging” model (Fig. 6B4) supposes that a specific protein or protein complex connects the sister chromosomes in the terminus region. Protein bridging between DNA molecules is known to contribute to chromosome organization and compaction through the action of proteins that include H-NS in E. coli, HU in B. subtilis, SMC proteins, MatP at the terminus in E. coli, and Spo0J at the origin in B. subtilis (73–78).

Regardless of which mechanism is employed, the fact that two distinct terminus foci could often be detected prior to and during chromosome translocation suggests that the site of cohesion is not at 171°, where the array was located, unless the cohesion site was able to slide on the chromosome, which is likely if cohesion is achieved through catenation. Further analysis of the distance between the two foci before and during translocation may shed light on the question whether the cohesion site is fixed.

It is not clear whether terminus cohesion has any role to play in sporulation. In mutants of *spo0A*, the master regulator for entry into sporulation, the sister chromosomes are known to become separated in a manner similar to vegetatively growing cells, and septation occurs at midcell instead of asymmetrically (2, 79). One plausible explanation would be that connection of the two chromosomes at midcell helps to block, through a nucleoid occlusion mechanism, medial division that would normally occur in vegetative cells (80–84). Formation of the asymmetric septum leads to the activation of σ^F^ in the prespore, specifically, and also generates a transient genetic asymmetry because the small prespore initially traps only one-third of a chromosome. While translocation of the remaining two-thirds of the chromosome into the prespore soon reestablishes genetic symmetry, the transient asymmetry is believed to be important for establishing different programs of transcription in the prespore and the mother cell (85, 86). The first important consequence is the activation of the mother cell-specific sigma factor (σ^E^), which is required for inhibiting the formation of a second asymmetric septum at the prespore-distal pole (45). This inhibitory effect is very sensitive to the timing of σ^E^ activation (48). Interestingly, it has further been observed that about 9% of the wild-type sporulating cells actually initiate a second septation event at the prespore-distal pole, but these secondary septa later disappeared (87). It is therefore possible that, in the event of second asymmetric septation, cohesion could delay the translocation of the mother cell chromosome into the second prespore, thereby preventing the formation of disporic cells, which cannot develop into robust endospores. Arriving at a better understanding of the basis for terminus cohesion may enable us to test whether it is required for efficient sporulation and, if so, to determine further specifics of its role.

## MATERIALS AND METHODS

### Bacterial growth.

For sporulation, B. subtilis cultures were inoculated from fresh plates and grown overnight in casein hydrolysate (CH) growth medium at 30°C with the addition of chloramphenicol (5 μg/ml). The following day, cultures were diluted to an optical density (*A*_600_) of 0.1 in 10 ml CH medium and grown at 37°C to an optical density (*A*_600_) of 0.8. Then, cultures were pelleted and resuspended into sporulation medium (SM) and grown for 1 h at 30°C, prior to time-lapse imaging (54, 88, 89). The time point of resuspension in SM is defined as *t*_0_, with subsequent time points in sporulation annotated likewise, measured in hours (i.e., *t*_1_ is 1 h after resuspension).

Strains of B. subtilis and E. coli were maintained on nutrient agar plates for short time periods. For transformation of B. subtilis, cells were grown in pretransformation medium (PTM) at 37°C to an optical density (*A*_600_) of approximately 3. Then, cells were added to DNA in transformation medium (TM) and grown for 1 h at 37°C before plating on suitable antibiotic-containing nutrient agar plates.

### Strain construction.

The “*ter* localization strain” was constructed to allow integration of the *tetO* array at 171° on the B. subtilis chromosome. For this, a region of the chromosome at 171° was amplified using the primers oCRW169 and oCRW170 (see Table S2 in the supplemental material) and inserted into plasmid pLau44-cat (90), which contained approximately 240 copies of the *tetO* array, at the BsrGI and EcoRV restriction enzyme sites.

The “−*7°-tetR-mCh”* and “7°-*tetR-gfp*” constructs were created using an overlapping PCR method. Upstream and downstream fragments were amplified by PCR, alongside amplification of the *tetR*, *mCh*, and *gfp* genes. The primers used are given in Tables S1 and S2 in the supplemental material. Primers contained overhanging bases that annealed to neighboring fragments. The forward primers for *mCh* and *gfp* also contained the P*_spoIIQ_* promoter sequence. Each amplified fragment was purified from an agarose gel using a gel extraction kit (Qiagen), and a final PCR step using 1 μl of each purified fragment was used to create a linear product, which was then transformed by B. subtilis.

### Time-lapse imaging using the CellASIC Onix microfluidic platform.

Time-lapse imaging was performed using the CellASIC Onix microfluidic platform (Merck Millipore/Merck). A Nikon Eclipse Ti inverted microscope equipped with a Nikon Plan Apo 100×/1.40 oil Ph3 objective using phase 3, a hot box, and a Rolera EM-C2 electron-multplying charge-coupled-device (EMCCD) camera was used for microscopy imaging. After resuspension into sporulation medium, cultures were incubated in flasks at 30°C for 1 h with shaking. Cells were then loaded to Onix B04A plates, which were sealed with a vacuum and incubated in a 32°C hot box. Sporulation medium (SM) containing CH was flowed at 2 lb/in^2^ for 2 h, followed by SM alone for the rest of the imaging. Imaging started 90 min after cells were loaded and typically proceeded every 3 min for 12 h, unless otherwise stated in the figure legend. Exposures of 100 ms were used for each channel.

FIJI ImageJ software (https://imagej.net/Fiji) was used for image processing and analysis (91). Each set of images for each channel was loaded into a stack, and the background signal was subtracted and then amplified analogously for each set of images. The StackReg plug-in was used with rigid-body stabilization to stabilize each stack of images (https://imagej.net/StackReg). Images from each channel were then merged together, and kymograms for individual cells were built from these stacks. We use the term “kymogram” to describe our figures rather than “kymograph,” as historically a kymograph described the machine used to produce a kymogram. The Kymograph Builder plug-in was used (http://imagej.net/KymographBuilder). Kymograms were manually adjusted for brightness and contrast.

### qPCR.

For qPCR methods, see the work of Koh and Murray (53). Briefly, the QIAgility (Qiagen) robot and software were used for setting up reactions. The Luna Universal qPCR master mix (NEB) was used in reactions, utilizing SYBR green dye-based detection and quantitation. Each 20-μl reaction mixture contained 10 μl qPCR master mix and 2 μl each 10 μM primer and ∼120 ng genomic DNA. Every reaction was set up in triplicate, and the final data were summarized as a mean average. The Rotor-Gene Q PCR instrument (Qiagen) was used to carry out reactions, with the accompanying software used to analyze results. The PCR was as follows: initial denaturation at 95°C for 3 min, followed by 40 cycles of the two-step reaction of 95°C for 5 s and 60°C for 30 s. Following the 40 cycles, the temperature was increased from 60°C to 95°C in 1°C increments every 5 s in order to produce melt curves. From the fluorescent amplification curves that were produced from the 40 cycles, threshold cycle (*C_T_*) values were calculated. The *C_T_* values were processed using the calculation 1/(2*^CT^*) to make the resulting values more biologically relevant.

## Supplementary Material

Supplemental file 1

Supplemental file 2

Supplemental file 3

Supplemental file 4

Supplemental file 5

Supplemental file 6

Supplemental file 7
