# Supplementary material for: Cohesion of Sister Chromosome Termini during the Early Stages of Sporulation in Bacillus subtilis
Source: J Bacteriol. 2020 Sep 23;202(20):e00296-20. doi: 10.1128/JB.00296-20 (PMC7515245; doi:10.1128/JB.00296-20)
Supplement: Supplemental file 1 [file JB.00296-20-s0001.pdf]

Table S1 Primers and templates used for making the *-7°-tetR-mCh and 7°-tetR-gfp* constructs

Construct	Fragment	Primers used	Template DNA
yxiF(-7°)- P_{spoIIQ}*-tetR-GFP-erm*	Up<br>P_{spoIIQ}*-tetR-GFP*<br>*erm*<br>Down	oCRW300, oCRW318<br>oCRW319, oCRW303<br>oCRW304, oCRW305<br>oCRW306, oCRW307	168CA gDNA<br>pWX510-GFP<br>pAPNC213-erm<br>168CA gDNA
yxiF(-7°)- P_{spoIIQ}*-tetR-mCh-erm*	Up<br>P_{spoIIQ}*-tetR-mCh*<br>*erm*<br>Down	oCRW300, oCRW318<br>oCRW319, oCRW371<br>oCRW372, oCRW305<br>oCRW306, oCRW307	168CA gDNA<br>pWX510<br>pAPNC213-erm<br>168CA gDNA

Table S2 Primers used in this study

Primer No	Sequence	Comments
oCRW169	ACGACTGTACATCTCCGCTCTAACAGCGTCTC	Classical cloning to create pLau44-dacC
oCRW170	AGACTGATATCGTTATCGACAGCGGAATTG	Classical cloning to create pLau44-dacC
oCRW300	GAACTGTTTCAGCTTCTATAGCAATCAGC	Overlap PCR to create *yxiF(-7°)-P_{spoIIQ}-tetR-GFP-erm* and *yxiF(-7°)-P_{spoIIQ}-tetR-mCh-erm*
oCRW303	GTTGCGCTCACTGCCCGCTTTCCAGTCGGGTT ATTTGTATAGTTCATCCATGC	Overlap PCR to create *yxiF(-7°)-P_{spoIIQ}-tetR-GFP-erm*
oCRW304	GATTACACATGGCATGGATGAACTATACAAAT AACCCGACTGGAAAGCGGGCAGTG	Overlap PCR to create *yxiF(-7°)-P_{spoIIQ}-tetR-GFP-erm*
oCRW305	GCATTGGATAAAAATGGGGCATTTATTGATGG AACGACTCATAGAATTATTTCCTC	Overlap PCR to create *yxiF(-7°)-P_{spoIIQ}-tetR-GFP-erm* and *yxiF(-7°)-P_{spoIIQ}-tetR-mCh-erm*
oCRW306	TTAACGGGAGGAAATAATTCTATGAGTCGTTC CATCAATAAATGCCCCATTTTTATCC	Overlap PCR to create *yxiF(-7°)-P_{spoIIQ}-tetR-GFP-erm* and *yxiF(-7°)-P_{spoIIQ}-tetR-mCh-erm*
oCRW307	GCGAAGACAAGCCATTCGCAACATATAAGTAT G	Overlap PCR to create *yxiF(-7°)-P_{spoIIQ}-tetR-GFP-erm* and *yxiF(-7°)-P_{spoIIQ}-tetR-mCh-erm*
oCRW318	CACTTTTCTGAAAATATATACAACCTATCCAC ATTAAATAAGAGATGTTACCATCC	Overlap PCR to create *yxiF(-7°)-P_{spoIIQ}-tetR-GFP-erm* and *yxiF(-7°)-P_{spoIIQ}-tetR-mCh-erm*
oCRW319	GAGAGGATGGTAACATCTCTTATTTAATGTGG ATAGGTTGTATATATTTTCAGAAAAGTGTTCA GAATGTTGCTGAGGGAGGGAGACGATTTTGAT GTCTAGATTAGATAAAAGTAAAG	Overlap PCR to create *yxiF(-7°)-P_{spoIIQ}-tetR-GFP-erm* and *yxiF(-7°)-P_{spoIIQ}-tetR-mCh-erm*
oCRW371	GTTGCGCTCACTGCCCGCTTTCCAGTCGGGTT ACTTGTACAGCTCGTCCATG	Overlap PCR to create *yxiF(-7°)-P_{spoIIQ}-tetR-mCh-erm*
oCRW372	CACCGGCGGCATGGACGAGCTGTACAAGTAAC CCGACTGGAAAGCGGGCAGTG	Overlap PCR to create *yxiF(-7°)-P_{spoIIQ}-tetR-mCh-erm*
oCRW206	GATTTCTGGCGAATTGGAAG	qPCR primer for *ori* product
oCRW325	AGAAAACACCCGGTTCACAC	qPCR primer for *ter-172* product
oCRW326	AGAAGGGGCTAAGCTCCAG	qPCR primer for *ter-172* product
oCRW344	GAATTCCTTCAGGCCATTGA	qPCR primer for *ori* product
oCRW345	TCCATATCCTCGCTCCTACG	qPCR primer for *ter-179* product
oCRW346	ATTCTGCTGATGTGCAATGG	qPCR primer for *ter-179* product
oCRW385	TCCCAATCGGAAAATCGCCA	qPCR primer for *ter-166*product
oCRW386	AGCAGCAGCAGCAATCTACA	qPCR primer for *ter-166*product

Figure S1: Characterisation of a newly constructed *tetOR-mCh* strain.

(A) Growth curves for CRW1 (168CA; white circles), CRW309 (*tetOR-mCh*; black triangles) and CRW593 (*tetR-mCh*; grey squares) grown in CH media at 37°C for 8 hours. Each growth curve represents the average growth of 3 cultures. (B) Box plots indicating cell length at t_0 for strains CRW1 (168CA, n=147 cells), CRW447 (*tetOR-mCh WALP23-gfp*, n=152 cells) and CRW148 (*rtp-gfp*, n=130 cells). (C) Distribution of cell length measurements and corresponding number of terminus foci for CRW447 (*tetOR-mCh WALP23-gfp*, n=152 cells) at t_0; cells with 1 focus (grey) and 2 foci (blue).

Figure S2: Snapshots of terminus movement within 9 sporulating cells from time lapse imaging.

Static images of 9 cells taken from time lapse experiments, imaging every 3 minutes, of strain CRW447 (*tetOR-mCh WALP23-gfp*), during sporulation. Each image is an overlay of the red (terminus) and green (membrane) channels. The first time point of each image is given and each subsequent image is 15 minutes after the previous one. Cells 1-4 and 5-9 are from two different, independent experiments. Scale bar is 1 µm.

Figure S3: Kymograms of terminus movement within 9 cells from time lapse imaging.

Kymograms are shown for 9 sporulating cells (identical to those in figure S2) taken from time lapse images of strain CRW447 (*tetOR-mCh WALP23-gfp*) using a microfluidic system, with 3 minute imaging intervals, between t_3-t_8 . Each kymogram is an overlay of the red (terminus) and green (membrane) kymograms for each cell, as indicated by the schematic. The points of asymmetric septation (green arrows) and terminus translocation (red arrows) are shown. Cells 1-4 and cells 5-9 are from different, independent experiments.

Figure S4: Localisation of RTP-GFP during sporulation.

Imaging of CRW148 (*rtp-gfp)* during sporulation at t_1, $t_{1.5}$ and t_2. (A) Diagrams depicting stages of sporulation (as defined in (1, 2))with DNA indicated in blue and RTP-GFP foci indicated in green. For each stage, localisation patterns for 1 terminus focus (left) and for 2 terminus foci (right) are shown. Coloured triangles indicate corresponding cells of each pattern in (B). (B) Images for phase contrast, a DAPI DNA stain, an FM5-95 membrane strain, RTP-GFP terminus foci and a merged image of membrane (red) and RTP-GFP (green) are

shown. 3 seconds exposure was used for the GFP channel. Coloured arrows indicate the stage of sporulation and the number of terminus, which correspond to the diagrams in (A). Images were processed with background and contrast adjustments. Scale bar is 3 µm.

Figure S5: Terminus movement imaged at 90 second intervals.

Static images of a sporulating cell taken from a time lapse experiment, imaged every 90 seconds, of strain CRW447 (*tetOR-mCh WALP23-gfp*). Each image is an overlay of the red (terminus) and green (membrane) channels. The frames shown are from $t_{2.725}$-$t_{3.3}$, 2 frames before and 21 frames after asymmetric septation.

Figure S6: Time lapse imaging of the chromosomal termini in a *spoIIIE36* mutant strain.

Time lapse imaging with 3 minute imaging intervals of strain CRW594 (*tetOR-mCh WALP23-gfp spoIIIE36*) was carried out in the microfluidic system during sporulation. (A) Static images of a representative cell from this time lapse, with each image 15 minutes after the previous image. Each image is an overlay of the red (terminus) and green (membrane) channels. Scale bar is 1 µm. (B) Kymograms of the representative cell shown in A, between t_3-$t_{6.5}$, showing red (terminus) and green (membrane) channels, alongside a merge of both kymograms. See corresponding movie S3.

Figure S7 Visualisation of the replication machinery in sporulating *B. subtilis* cells.

A *B. subtilis* strain (CRW551) containing WALP-GFP (green), a 171°-*tetO* array bound by TetR-mCh (red) and a GFP-DnaN (green) was imaged every 3 minutes using microfluidic time lapse imaging. Kymograms of a single cell from this strain are shown for a 10 hour period, where the timings are relative to sporulation resuspension. Kymograms are shown for the red channel only (left), green channel only (centre) and a composite overlay kymogram (right), as indicated by the cartoons. The white line indicates where the cell is still growing and rotating in the early stages of the kymogram. The red arrow shows the peak of termini movement towards the septum, the green arrow shows the formation of the septum and the yellow arrow marks the disappearance of the GFP-DnaN focus.

Figure S8 Measurement of the timing of cellular events during sporulation from a time lapse of a DnaN-GFP strain.

A time lapse of CRW551 (*tetOR-mCh WALP23-gfp dnaN-gfp*) after sporulation initiation was carried out, imaging every 3 minutes using the microfluidic system. In 20 cells, the time points at which a DnaN-GFP focus dissociated, the asymmetric septum formed and the terminus had completed movement towards the septum were counted as measures of replication completion, septation and terminus translocation, respectively. The time between these events are plotted in the box plots, showing each measurement for each cell as blue diamonds and the quartile and total range indicated in the box plot.

Movie legends

Movie S1: Time lapse imaging of *ter* localisation in a wild type strain during sporulation. Movie of a representative cell from a time lapse of strain CRW447 (*tetOR-mCh WALP23-gfp*; wild type) between T_3-T_8 hours of sporulation, corresponding to kymogram in Fig. 1. Red (terminus TetR-mCh) and green (membrane WALP-GFP) signals are overlayed. Scale bar is 1 µm.

Movie S2: Time lapse imaging with 90 second intervals to follow terminus movement in a wild type strain. Movie of a representative cell from a time lapse of strain CRW447 (*tetOR-mCh WALP23-gfp*; wild type) between T_2-$T_{5.5}$ hours of sporulation, imaging every 90 seconds, corresponding to kymogram in Fig. 2A. Red (terminus TetR-mCh) and green (membrane WALP-GFP) signals are overlayed. Scale bar is 1 µm.

Movie S3: Time lapse imaging of the chromosomal termini in a *spoIIIE36* mutant strain. Movie of a representative cell from a time lapse of strain CRW594 (*tetOR-mCh WALP23-gfp spoIIIE36*) between T_3-T_8 hours of sporulation, corresponding to static images and kymographs in Fig. S6. Red (terminus TetR-mCh) and green (membrane WALP-GFP) signals are overlayed. Scale bar is 1 µm.

Movie S4: Time lapse imaging to show terminus translocation to the prespore using a *-7°-tetR-mCh* construct. Movie of a representative cell from a time lapse of strain CRW540 (*tetOR-mCh WALP23-gfp -7°-tetR-mCh* between T_3-T_8 hours of sporulation, corresponding to static images and kymograms in Fig. 3. Red (prespore and mother cell terminus TetR-mCh) and green (membrane WALP-GFP) signals are overlayed. Scale bar is 1 µm.

Movie S5: Time lapse imaging to show terminus translocation to the prespore using a *-7°-tetR-gfp* construct. Movie of a representative cell from a time lapse of strain CRW509 (*tetOR-mCh WALP23-gfp -7°-tetR-gfp* between T_3-T_8 hours of sporulation, corresponding to static images and

kymograms in Fig. 3. Red (terminus TetR-mCh) and green (membrane WALP-GFP and prespore TetR-mCh) signals are overlayed. Scale bar is 1 µm.

Movie S6: Time lapse imaging to show terminus movement in a *spoIIGA* mutant. Movie of a representative cell from a time lapse of strain CRW595 (*tetOR-mCh WALP23-gfp spoIIGA*) between T_3-T_8 hours of sporulation, corresponding to kymograms in Fig. 4. Red (terminus TetR-mCh) and green (membrane WALP-GFP) signals are overlayed. Scale bar is 1 µm.

REFERENCES

1. Bogush M, Xenopoulos P, Piggot PJ. 2007. Separation of Chromosome Termini during Sporulation of *Bacillus subtilis* Depends on SpoIIIE. Journal of Bacteriology 189:3564-3572.
2. Illing N, Errington J. 1991. Genetic regulation of morphogenesis in *Bacillus subtilis*: roles of sigma E and sigma F in prespore engulfment. Journal of Bacteriology 173:3159-3169.

Fig S1

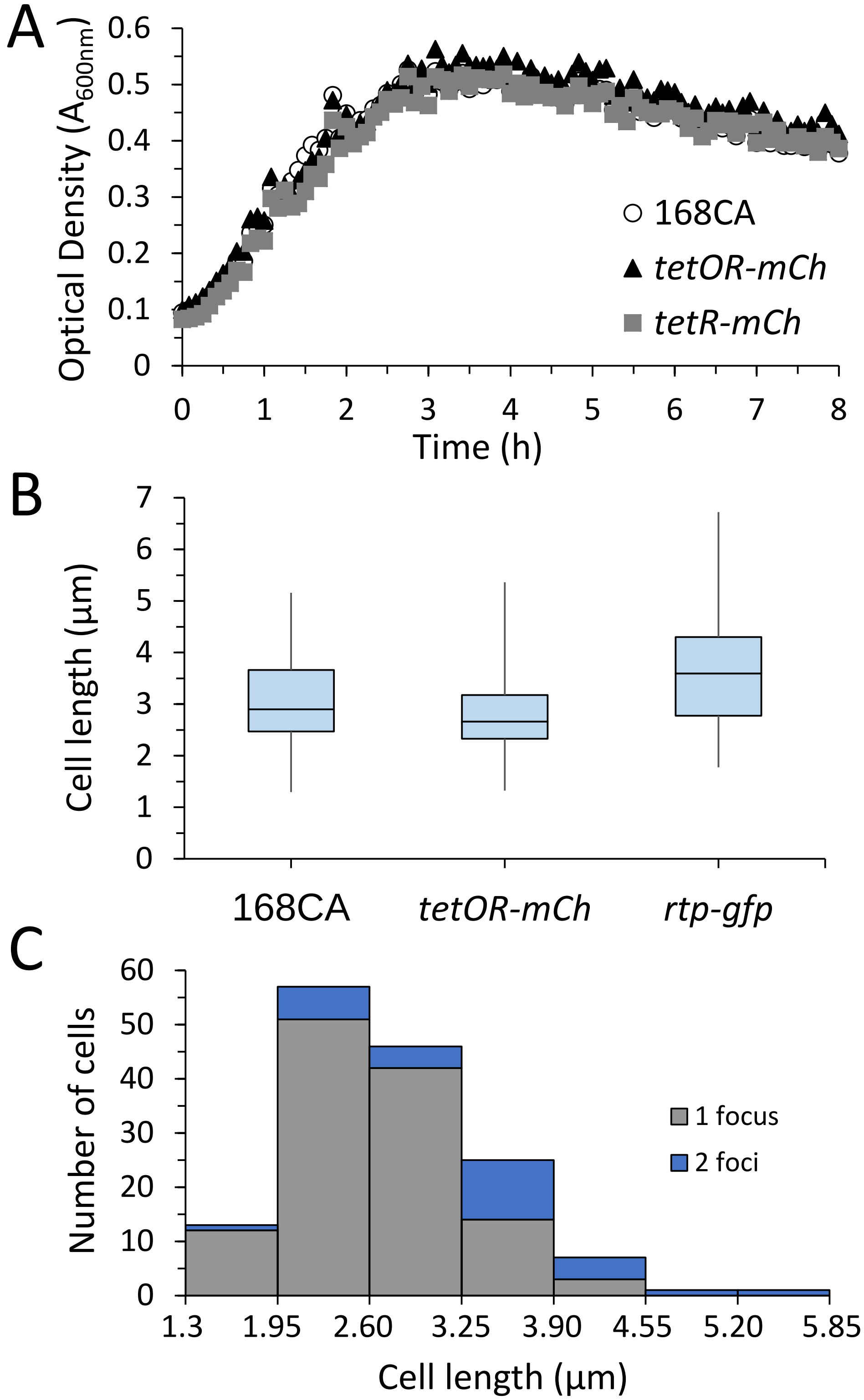
A
Optical Density (A600nm)
0.6
0.5
0.4
0.3
0.2
0.1
0
0 1 2 3 4 5 6 7 8
Time (h)
168CA
tetOR-mCh
tetR-mCh
B
Cell length (µm)
7
6
5
4
3
2
1
0
168CA
tetOR-mCh
rtp-gfp
C
Number of cells
60
50
40
30
20
10
0
1.3 1.95 2.60 3.25 3.90 4.55 5.20 5.85
Cell length (µm)
1 focus
2 foci

Fig S2

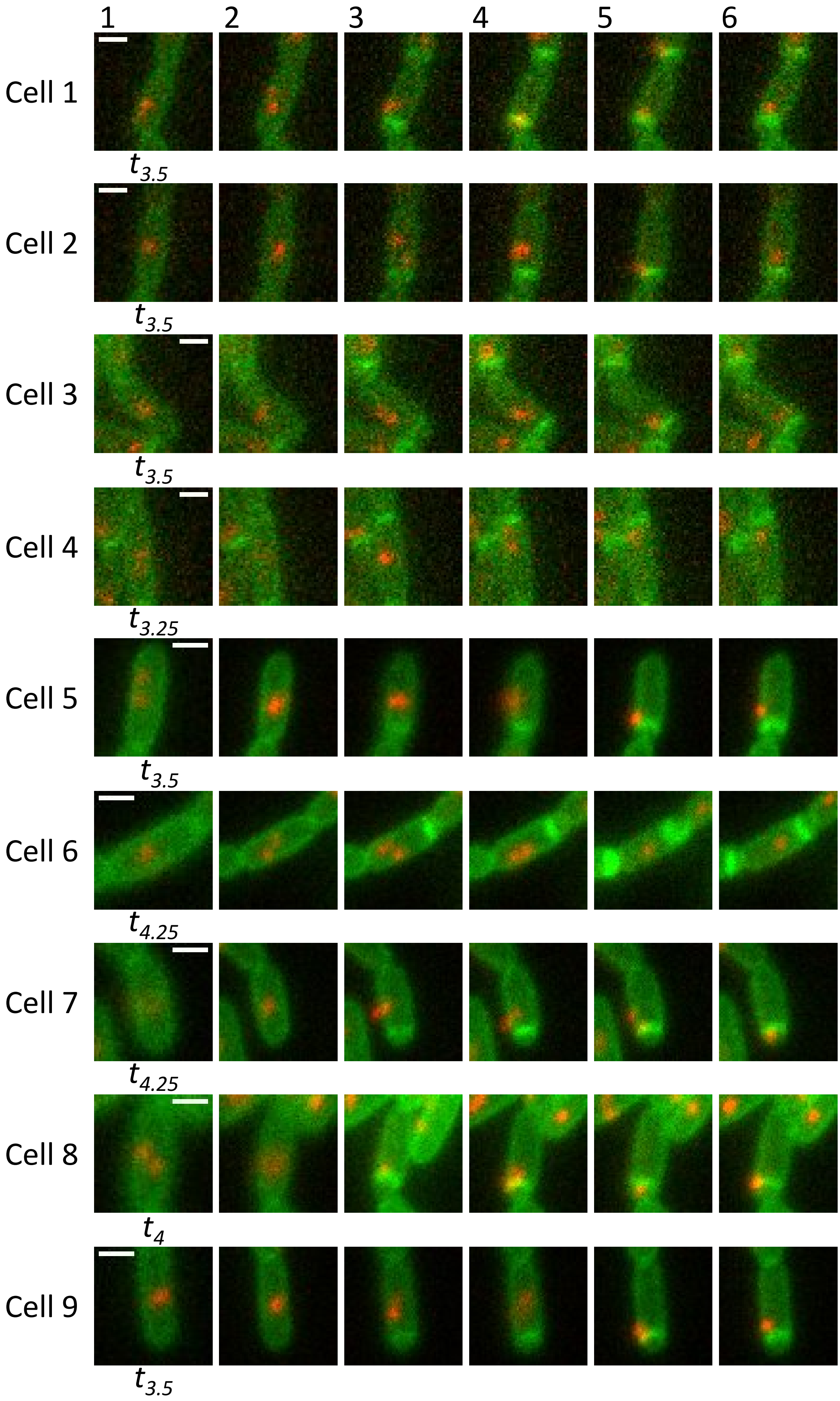
1
2
3
4
5
6
Cell 1
$t_{3.5}$
Cell 2
$t_{3.5}$
Cell 3
$t_{3.5}$
Cell 4
$t_{3.25}$
Cell 5
$t_{3.5}$
Cell 6
$t_{4.25}$
Cell 7
$t_{4.25}$
Cell 8
t_4
Cell 9
$t_{3.5}$

Fig S3

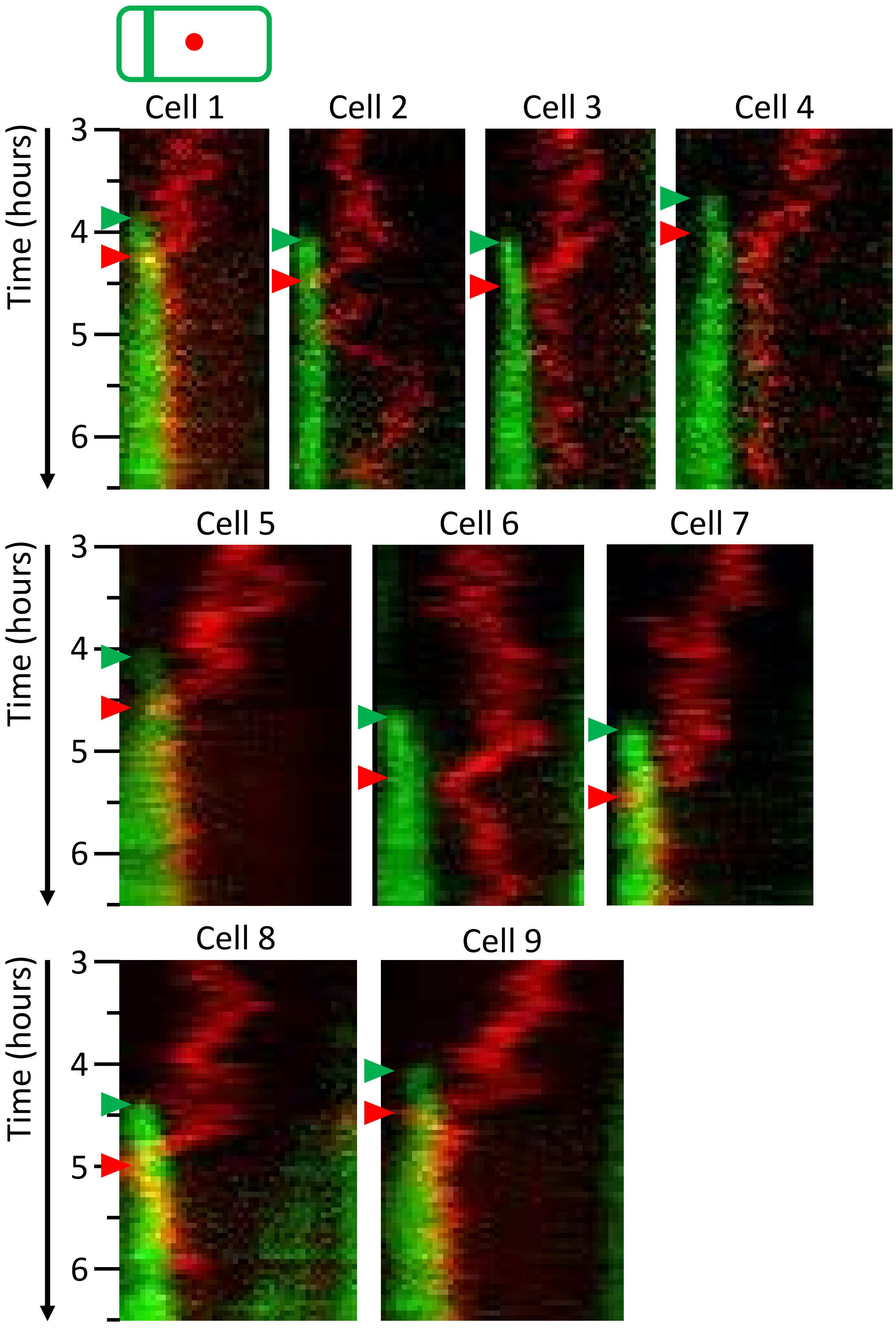
Cell 1
Cell 2
Cell 3
Cell 4
Cell 5
Cell 6
Cell 7
Cell 8
Cell 9
Time (hours)
3
4
5
6

Fig S4

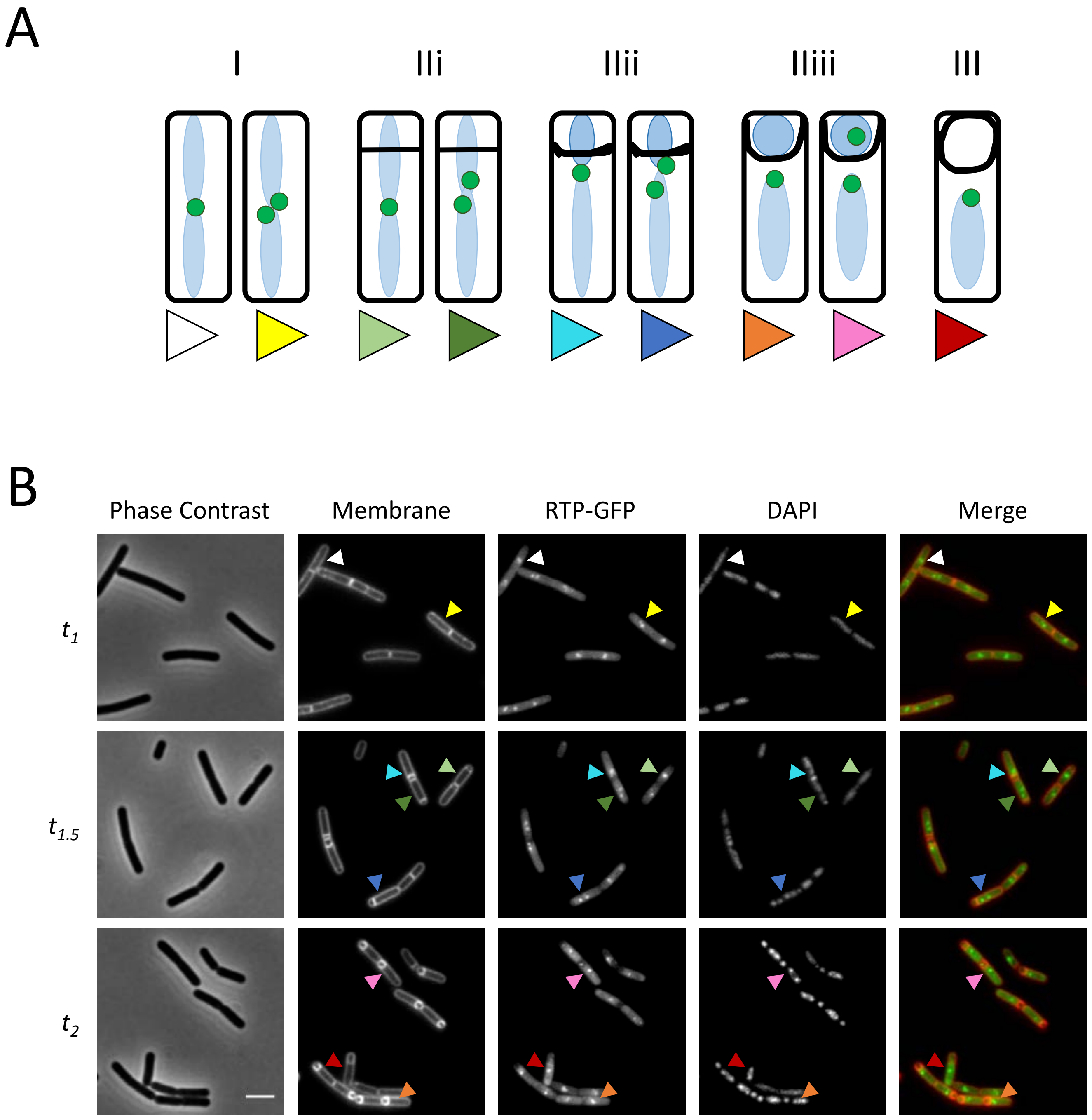
A
I
IIi
IIii
IIiii
III
B
Phase Contrast
Membrane
RTP-GFP
DAPI
Merge
t_1
$t_{1.5}$
t_2

Fig S5

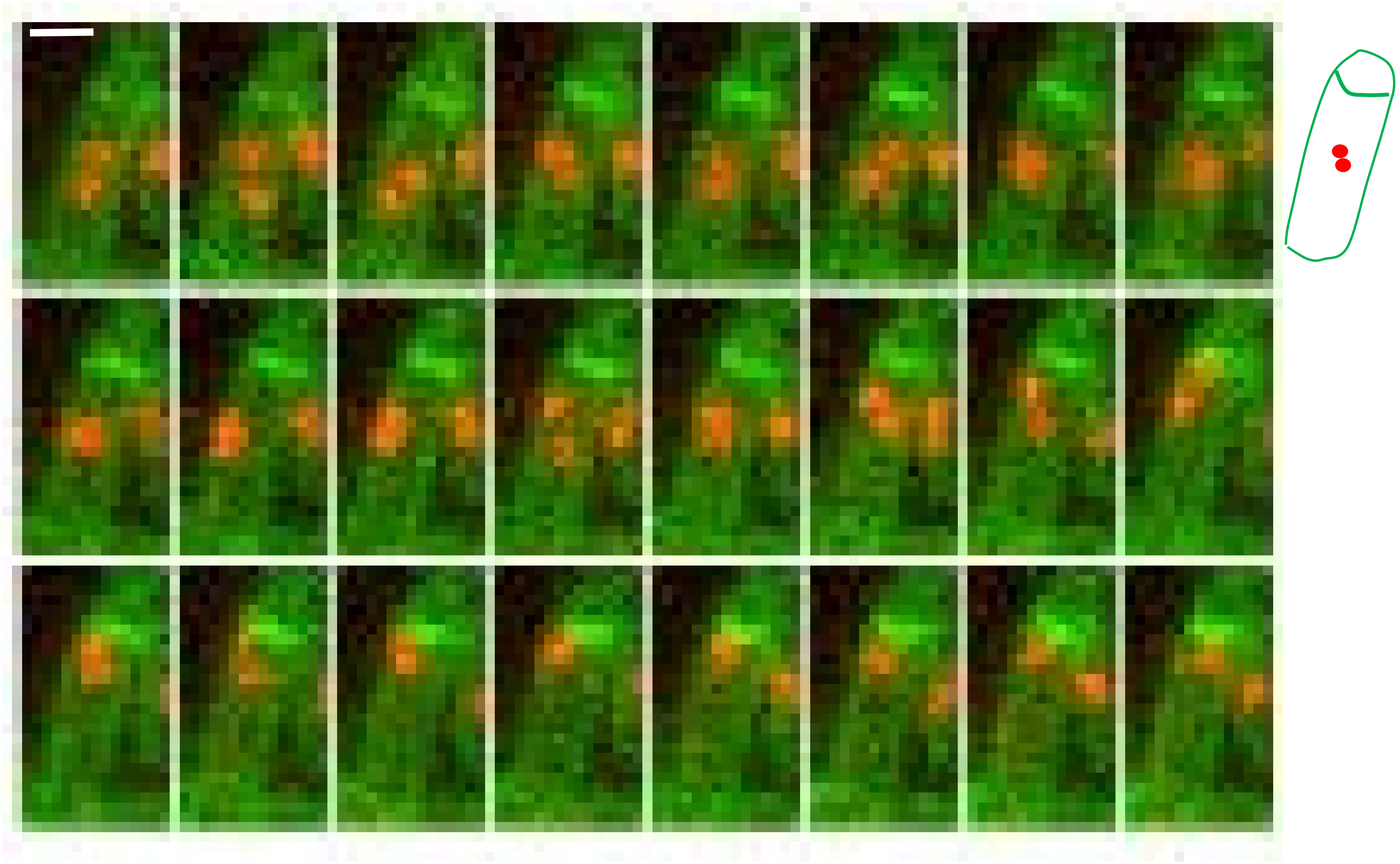

Fig S6

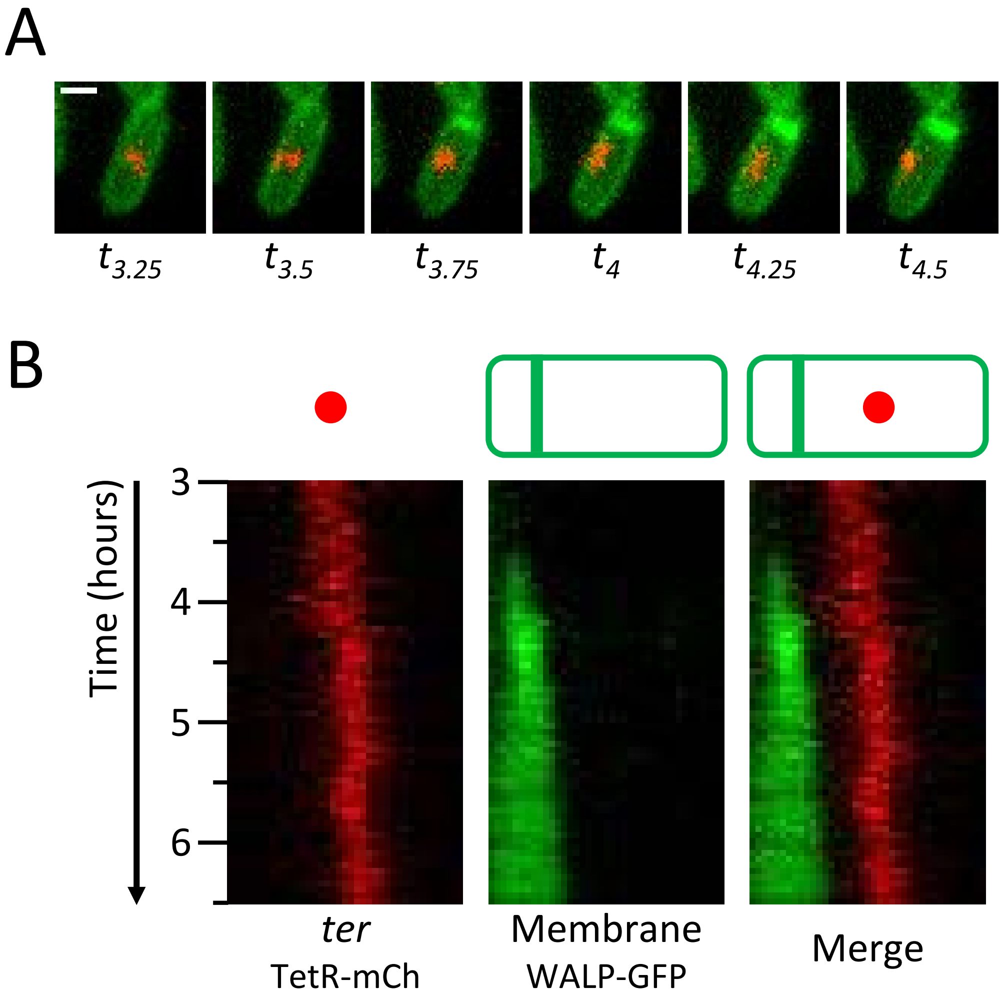
A
$t_{3.25}$
$t_{3.5}$
$t_{3.75}$
t_4
$t_{4.25}$
$t_{4.5}$
B
Time (hours)
3
4
5
6
ter
TetR-mCh
Membrane
WALP-GFP
Merge

Fig S7

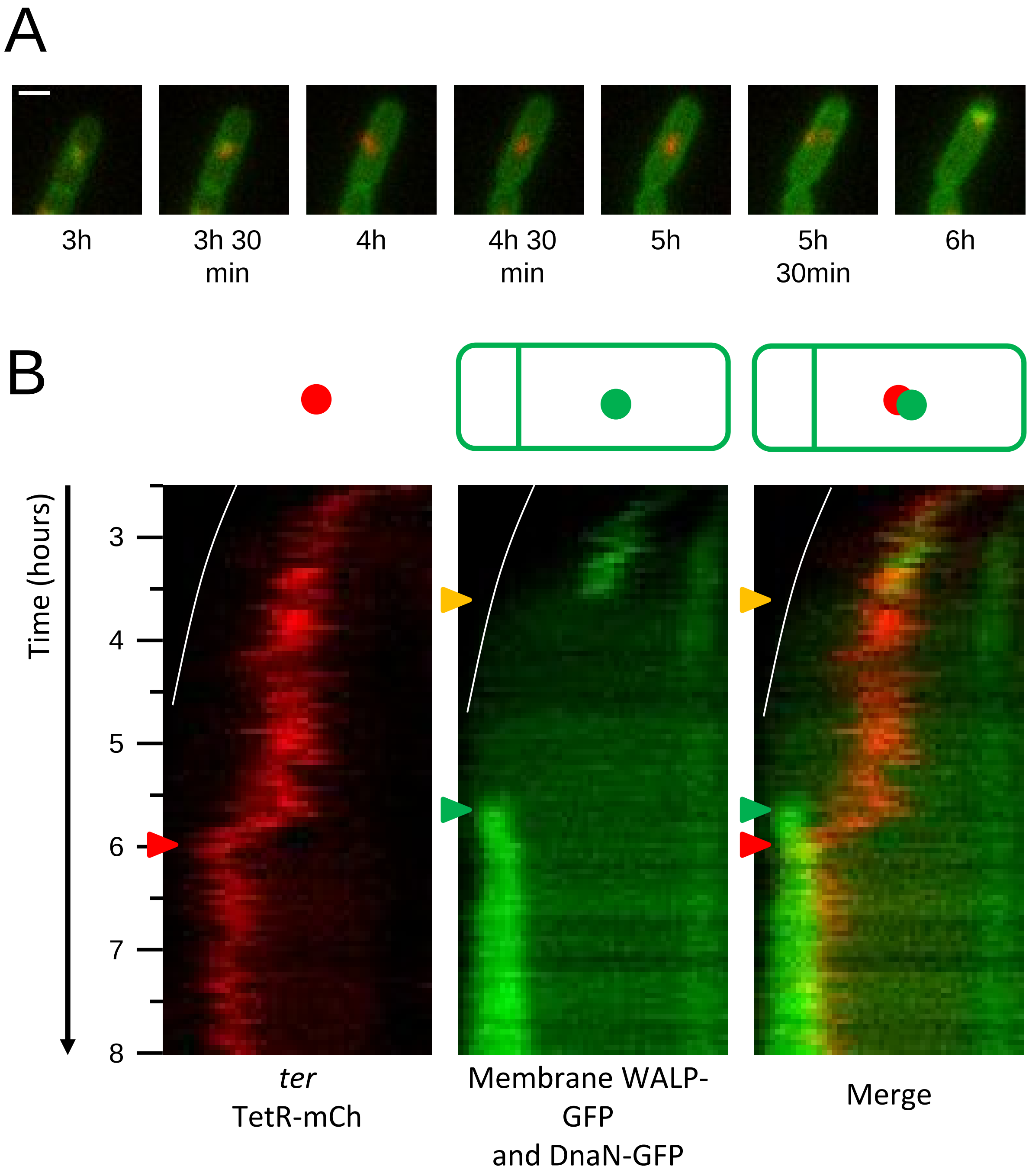
A
3h
3h 30 min
4h
4h 30 min
5h
5h 30min
6h
B
Time (hours)
3
4
5
6
7
8
ter
TetR-mCh
Membrane WALP-GFP
and DnaN-GFP
Merge

Fig S8

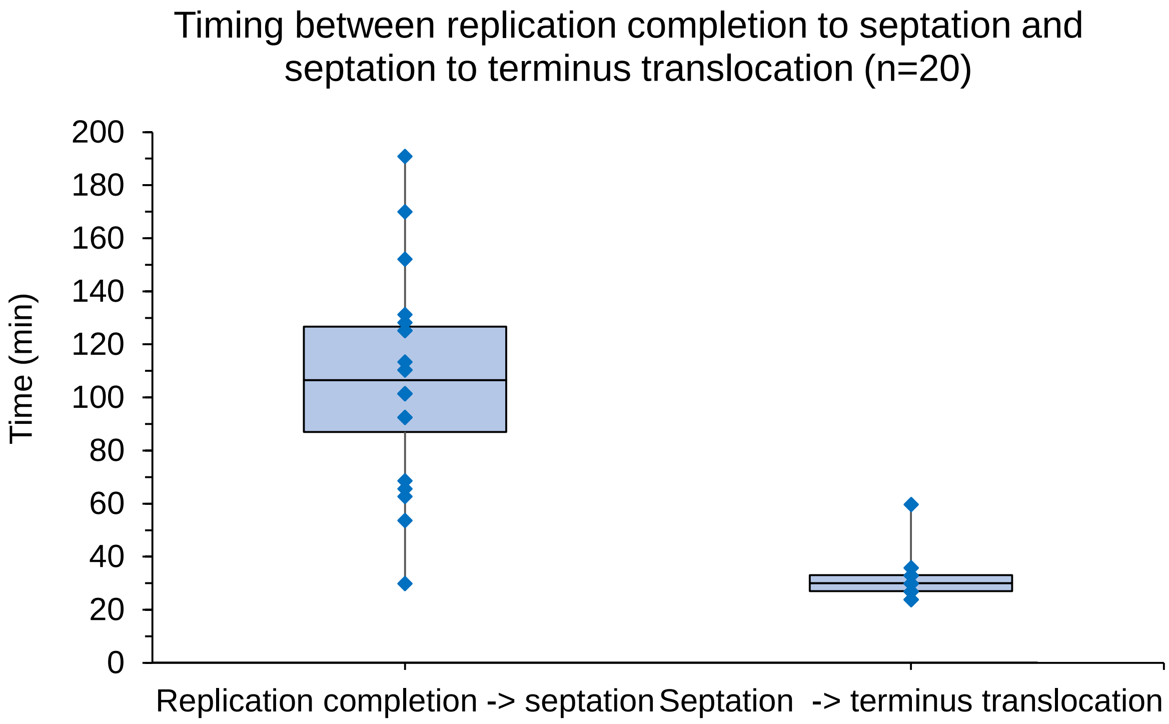

Timing between replication completion to septation and septation to terminus translocation (n=20)
Time (min)
200
180
160
140
120
100
80
60
40
20
0
Replication completion -> septation
Septation -> terminus translocation
